# Exploring the Role of Advanced Composites and Biocomposites in Agricultural Machinery and Equipment: Insights into Design, Performance, and Sustainability

**DOI:** 10.3390/polym17121691

**Published:** 2025-06-18

**Authors:** Ehsan Fartash Naeimi, Kemal Çağatay Selvi, Nicoleta Ungureanu

**Affiliations:** 1 Department of Agricultural Machinery and Technologies Engineering, Faculty of Agriculture, Ondokuz Mayis University, 55139 Samsun, Türkiye; agri.ehsan@gmail.com; 2 Department of Biotechnical Systems, Faculty of Biotechnical Systems Engineering, National University of Science and Technology Politehnica Bucharest, 060042 Bucharest, Romania

**Keywords:** natural fibers, agricultural equipment design, fiber-reinforced polymers, eco-efficient materials, polymer matrix composite

## Abstract

The agricultural sector faces growing pressure to enhance productivity and sustainability, prompting innovation in machinery design. Traditional materials such as steel still dominate but are a cause of increased weight, soil compaction, increased fuel consumption, and corrosion. Composite materials—and, more specifically, fiber-reinforced polymers (FRPs)—offer appealing alternatives due to their high specific strength and stiffness, corrosion resistance, and design flexibility. Meanwhile, increasing environmental awareness has triggered interest in biocomposites, which contain natural fibers (e.g., flax, hemp, straw) and/or bio-based resins (e.g., PLA, biopolyesters), aligned with circular economy principles. This review offers a comprehensive overview of synthetic composites and biocomposites for agricultural machinery and equipment (AME). It briefly presents their fundamental constituents—fibers, matrices, and fillers—and recapitulates relevant mechanical and environmental properties. Key manufacturing processes such as hand lay-up, compression molding, resin transfer molding (RTM), pultrusion, and injection molding are discussed in terms of their applicability, benefits, and limits for the manufacture of AME. Current applications in tractors, sprayers, harvesters, and planters are covered in the article, with advantages such as lightweighting, corrosion resistance, flexibility and sustainability. Challenges are also reviewed, including the cost, repairability of damage, and end-of-life (EoL) issues for composites and the moisture sensitivity, performance variation, and standardization for biocomposites. Finally, principal research needs are outlined, including material development, long-term performance testing, sustainable and scalable production, recycling, and the development of industry-specific standards. This synthesis is a practical guide for researchers, engineers, and manufacturers who want to introduce innovative material solutions for more efficient, longer lasting, and more sustainable agricultural machinery.

## 1. Introduction

Agriculture is a pillar of the global economy and food security, but it operates under immense pressure to increase production while minimizing its environmental footprint [1]. Technological progress, particularly in agricultural machinery and equipment (AME), is key to achieving greater efficiency, precision, and productivity in farm operations [2,3]. Over the last decades, the production of tractors, combines, sprayers, planters, and tillage equipment has been predominantly ferrous metal (cast iron, steel) and aluminum alloy based. These possess adequate strength, stiffness, and manufacturability at relatively low costs [4]. However, their high weight is a major drawback. Heavy machinery compacts the soil, which negatively affects soil structure, water infiltration, and root growth, ultimately leading to reduced crop yields [5,6]. In addition, the heavy metal equipment weighs more, necessitating more powerful engines, leading to higher fuel consumption and associated greenhouse gas emissions [7]. Metallic components are also vulnerable to corrosion by water, soil, fertilizers, and pesticides, which demand protective coats and regular maintenance, thus increasing lifetime costs [8]. The reduction of these drawbacks by upscale material integration becomes increasingly significant for effective and sustainable farming practices.

As an answer to these challenges, composite materials have emerged as promising alternatives. For example, fiber-reinforced polymers (FRPs), tough fibers suspended in a polymer matrix, offer a paradigm shift in material selection for agricultural machinery [9]. High strength-to-weight and stiffness-to-weight ratios, good corrosion and chemical attack resistance, acceptable fatigue characteristics, and significant design flexibility for developing integrated and intricate structures are some of the key advantages of FRPs [10,11]. Direct employment of such materials eliminates the main drawbacks of common metals. In the context of sustainable and efficient farming practice, the incorporation of advanced materials in equipment design has become more and more indispensable.

The new world trend towards circular economy, resource efficiency, and sustainability has increased the focus on biocomposite materials [12,13]. Biocomposites have been defined as composite materials in which one or more of the constituent phases (reinforcement or matrix) are of biological origin [14]. Typically, they incorporate natural fibers—such as flax, hemp, jute, kenaf, sisal, or even crop residues including straw—as reinforcement, either with traditional petroleum-based polymers or increasingly, bio-based polymers from renewable feedstocks (e.g., corn, sugarcane, vegetable oils) [15,16]. Natural fibers have advantages such as low density, renewability, potential biodegradability, reduced environmental impact in manufacture (compared to glass fiber), reduced abrasiveness to machinery, and advantageous specific mechanical characteristics [17,18]. The application of these materials is therefore in harmony with the increasing focus on sustainable, bio-based systems in the agriculture industry.

The application of composites and biocomposites in agricultural machinery has the potential to revolutionize equipment performance by offering lighter, fuel-efficient, longer-lived, and eco-friendly alternatives [19,20]. However, several technical complexities, cost factors, and long-term durability issues under harsh agricultural conditions must be addressed. These have limited the extensive application of composite and biocomposite materials in agricultural machinery versus other sectors.

The present review aims to provide a critical and integrative overview of the current trends and perspectives of composite and biocomposite materials for farm machinery. Based on recent peer-reviewed publications, this review critically examines material definitions, classifications, production methods, specific applications, comparative advantages, inherent challenges, and potential research areas in the future. The objective is to bridge existing knowledge, identify critical gaps, and enable the development of new and effective uses of advanced materials in agriculture.

## 2. Background and Definitions

Understanding the intrinsic nature of composites and biocomposites, including their constituent material and intrinsic properties, is a precursor to an understanding of their specific applications and limitations in the demanding environment of AME. The unique operating conditions of AME—such as exposure to harsh weather, soil exposure, corrosive agrochemicals, dynamic mechanical stresses, and increasing pressure for operational efficiency and sustainability—place specific demands on material performance that composites and biocomposites aim to fulfill.

### 2.1. Definition of Composites

A composite material is an engineered, or in some cases, a naturally occurring material, created by combining two or more distinct constituents with significantly different physical or chemical properties. A key characteristic of composites is that these components remain separate and identifiable within the final structure at either the macroscopic or microscopic level [10,21]. The primary objective of combining these components is to achieve a synergistic effect—producing a material with enhanced or unique properties such as high specific strength, stiffness, corrosion resistance, or tailored thermal and electrical characteristics—that surpasses those of the individual constituents when used independently [11,20].

The basic architecture of composite material includes two primary elements: the matrix and the reinforcement. The matrix acts as the continuous phase, encapsulating and supporting the reinforcement, thereby maintaining the overall geometry and structural integrity of the composite component [22]. It also facilitates efficient load transfer to the typically stiffer and stronger reinforcement phase. Furthermore, the matrix plays a vital role in protecting the reinforcement from environmental degradation specific to agricultural settings, including moisture ingress from rain or soil, attack by fertilizers and pesticides, impact damage, and abrasion from dust and crop residues [4,20]. Depending on the application, matrices may be polymeric, metallic, or ceramic in nature; however, polymer matrix composites (PMCs) are most widely utilized in AME due to their corrosion resistance, light weight, and ability to be molded into complex geometries [23].

The reinforcement, which forms the dispersed phase, is primarily responsible for bearing mechanical loads encountered during AME operation (e.g., structural support, lifting, soil engagement) [18]. It can take the form of fibers (short or continuous), particles, or flakes. Among these, fiber-reinforced composites are especially common in AME applications due to their superior stiffness-to-weight and strength-to-weight ratios [20,24]. These properties are critical for reducing soil compaction, improving fuel efficiency, and enabling the design of larger and more effective implements, such as wider sprayer booms or high-capacity seeders. Synthetic fibers such as glass, carbon, and aramid are commonly used for their high tensile strength, predictable properties, and fatigue resistance under cyclic loads [25].

An essential scientific aspect of composite design, particularly for durable and reliable AME applications, is the interfacial adhesion between the matrix and the reinforcement [26,27]. Effective load transfer hinges on strong interfacial bonding, which governs the mechanical integrity, fatigue life, and environmental resistance of the composite under harsh operational conditions. Poor adhesion can lead to premature failure mechanisms such as fiber pull-out, delamination, or matrix cracking under operational stresses, significantly undermining component performance and longevity [26,28]. Especially in moist agricultural environments, a weak interface can provide pathways for moisture ingress, accelerating degradation. To address this, surface treatments applied to fibers, or the incorporation of specialized chemicals known as coupling agents and compatibilizers into the matrix formulation, are often employed to enhance chemical bonding and physical interlocking between the phases, ensuring robust performance under demanding AME conditions [19,27].

### 2.2. Definition of Biocomposites

Biocomposites represent a rapidly expanding class of composite materials distinguished by the utilization of one or more components derived from renewable biological sources. This differentiates them from conventional composites, whose primary application is based on petroleum-derived polymers and energy-intensive synthetic fibers [29,30]. As a more environmentally friendly alternative, biocomposites purposefully position themselves within global initiatives aimed at environmental conservation, reduced dependence on fossil fuels, and the implementation of circular economy concepts—concepts that are becoming more applicable in the agricultural sector, which is both a producer of biomass and a user of resource-intensive machinery [21].

Generally, biocomposites can be classified into three primary types based on their constituent materials.

#### 2.2.1. Natural Fiber-Reinforced Conventional (Petroleum-Based) Matrices

This is currently the most prevalent type of biocomposite researched for semi-structural use in numerous industries, such as AME. These biocomposites utilize plant-based reinforcements, such as flax, jute, hemp, kenaf, or even processed agricultural residues such as straw, embedded into proven synthetic resins such as polypropylene (PP), polyethylene (PE), or in some cases thermosets such as unsaturated polyesters. While the matrix remains petroleum-derived, the incorporation of renewable, low-density natural fibers significantly reduces the overall weight and dependence on synthetic reinforcements, thereby lowering the environmental impact compared to conventional composites such as glass fiber-reinforced polymer (GFRP) [31,32].

**Figure 1:**
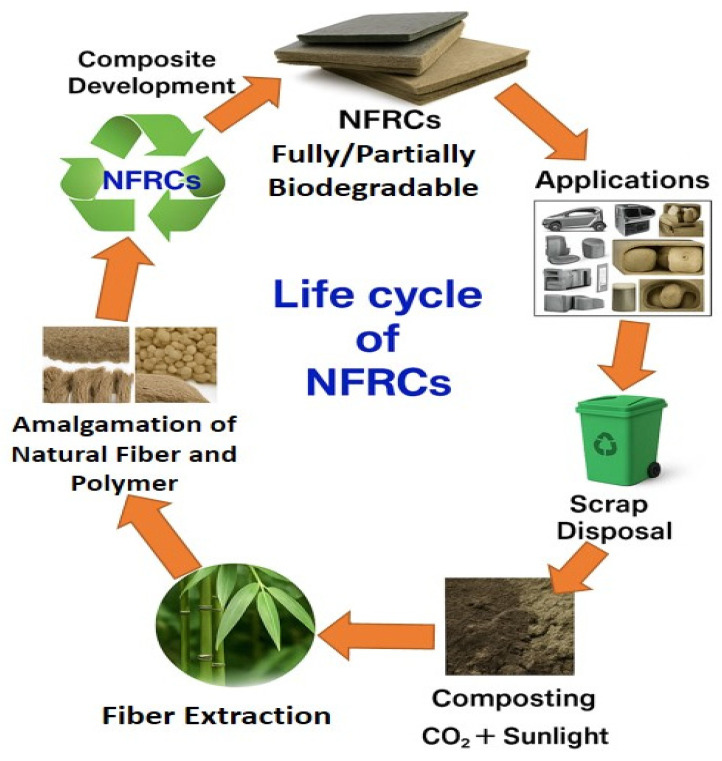
Life cycle of natural fiber-reinforced composites (NFRCs) (diagram drawn by the authors).

The life cycle of biodegradable natural fiber-reinforced composites (NFRCs) is depicted in Figure 1. Tractor interior panels, engine covers, and light protective guards are some applications of natural fiber-reinforced composites in AME.

#### 2.2.2. Synthetic Fiber-Reinforced Bio-Based Matrices

In this relatively uncommon configuration, conventional synthetic fibers (i.e., glass or, more unusually, carbon) provide the primary structural reinforcement, delivering elevated mechanical strength and stiffness. Meanwhile, the matrix is made up of polymers from renewable sources, such as polylactic acid (PLA), polyhydroxyalkanoates (PHA), or bio-based counterparts of conventional polymers such as Bio-PET or Bio-PE [33,34,35]. This approach seeks to lower the fossil-based carbon footprint of high-performance composites, but the non-biodegradability of synthetic fibers continues to be a limiting factor. Moreover, the relatively high cost and potentially inferior thermo-mechanical properties of bio-based resins compared to petrochemical counterparts presently restrict their widespread application in demanding AME components [31,36].

#### 2.2.3. Fully Bio-Based Composites (Green Composites)

These are the most environmentally friendly form of biocomposites, where both the reinforcement and the matrix are completely of biological and renewable origin. Examples include natural fibers such as hemp or flax fibers in biodegradable polymer matrices of PHA or PLA [37,38]. These types of materials have inherent compostability or biodegradability, offering end-of-life options that can be particularly useful for certain agricultural applications with limited-service life or direct contact with soil (e.g., biodegradable planting pots, mulch film components, specialized sensor housings). However, their application to durable AME components is currently restricted by enormous difficulties in mechanical performance (often lower strength and stiffness), thermal stability, and long-term durability, specifically in the context of moisture sensitivity [39].

The merits of biocomposites for AME go far beyond environmental sustainability. Natural fibers are low in density by nature, which helps in the design of lightweight components—a top priority for reducing soil compaction and fuel consumption in agricultural machinery and vehicles [40]. Natural fibers also tend to have good acoustic and thermal insulation, which can be utilized to enhance operator comfort in tractor cabins and protect sensitive electronics. Their lower abrasion compared to glass fibers also imposes less wear on the production equipment such as molds, dies, and cutters, which can translate into reduced overall production costs [41,42]. Nevertheless, several barriers must be addressed to facilitate broader adoption in AME. The hydrophilic nature of natural fibers leads to the uptake of moisture, which can cause swelling, dimensional instability, microbial degradation, and a dramatic loss in mechanical performance [42,43]. Furthermore, natural variation in the quality of fibers—based on species, harvest time, and processing methods—can potentially prevent the attainment of repeatable product performance, especially in comparison with synthetic reinforcements [44,45]. Another challenge is the weak interfacial bonding between the polar surface of natural fibers and the normally non-polar nature of polymer matrices, where chemical surface treatment or fiber hybridization approaches are necessary to increase the load transfer efficiency and long-term durability in dynamic or high-stress environments common for agricultural equipment [26,46].

**Figure 2:**
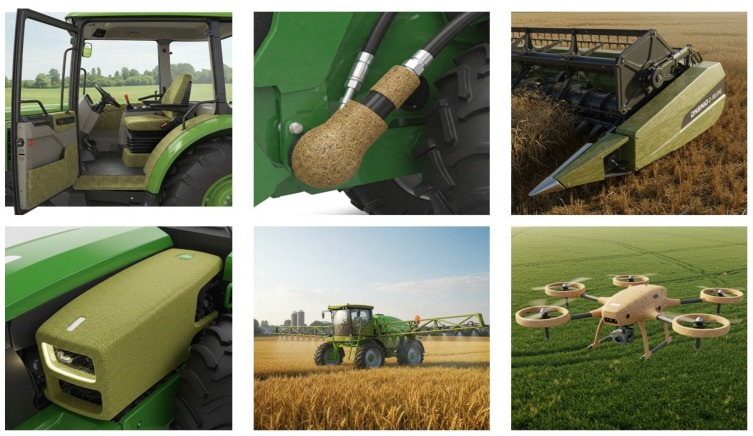
Examples of agricultural machinery and equipment incorporating components made from fully bio-based composites.

Recent advances in bio-based polymer chemistry—including the development of high-performance biopolymers such as bio-polyamides and high-performance bio-polyesters from agricultural or forestry residues—have dramatically expanded the number of possible applications for green composites [41,47,48]. In parallel, innovations in green manufacturing techniques, such as solvent-free processing, optimized thermo-compression cycles for natural fibers, and additive manufacturing (3D printing) using biocomposite filaments, are enhancing the energy efficiency and scalability of production methods [49,50,51]. While the current use of these kinds of materials in AME is largely limited to non-structural or semi-structural components—e.g., panels, fairings, interior components, and storage tanks for non-corrosive materials—ongoing research is committed to enhancing their mechanical and environmental durability, with the long-term goal of making them viable for more structurally demanding applications and enabling a more sustainable agricultural mechanization in the years to come [52,53,54]. In line with this, it is anticipated that in the future, as illustrated in Figure 2, most of the agricultural machinery and equipment components will be designed and manufactured using such materials wherever feasible.

## 3. Types of Materials Used in Composites and Biocomposites

The performance, durability, cost-effectiveness, and sustainability of composite and biocomposite materials are primarily determined by the nature and interaction of their constituent components—specifically the reinforcing fibers, polymer matrices, and fillers. The judicious selection and combination of these components enable engineers and researchers to tailor the mechanical, thermal, and environmental properties of composites to meet the specific demands of various applications, particularly in the agriculture and automotive sectors, where requirements often include durability, resistance to harsh environments, and increasingly, lightweighting for efficiency and reduced soil impact.

### 3.1. Reinforcing Fibers

**Figure 3:**
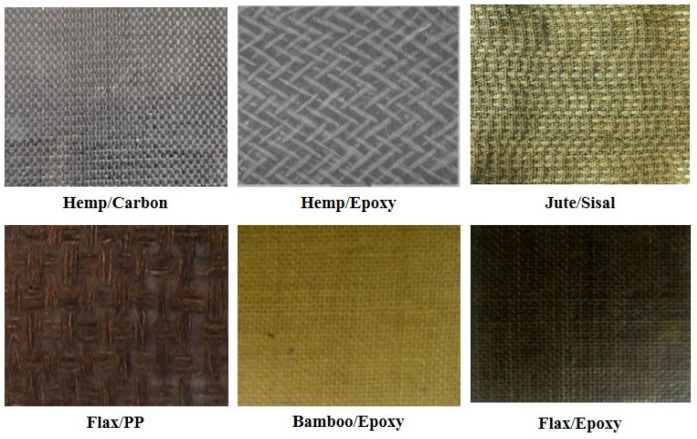
Examples of common reinforcing fibers used in composites, either in hybrid forms or in the presence of a polymer matrix, reprinted with permission from Elsevier [55].

Reinforcing fibers are responsible for determining the load-carrying capacity, stiffness, and overall mechanical strength of composite materials. Fibers can be broadly categorized into synthetic and natural types, both having advantages and disadvantages associated with them. The choice between them would depend on the specific requirements of the parts in agriculture machinery, such as durability, weight, and environmental considerations. These reinforcing fibers, either as hybrids or embedded within a polymer matrix, are exemplified by examples in Figure 3, indicating their application across various industries. Similar examples of these composites can be observed and analyzed in numerous studies [55,56,57,58].

#### 3.1.1. Synthetic Fibers

Synthetic fibers form the basis of conventional high-performance composites in a wide range of industries, including agriculture.

- Glass fibers: Among synthetic fibers, glass fibers—specifically E-glass (electrical grade)—are the most extensively used reinforcement due to their favorable combination of low cost, acceptable tensile strength, excellent electrical insulation, and corrosion resistance [59,60]. S-glass, which is more expensive, has superior mechanical performance (higher strength and stiffness) and can be utilized in niche agrarian uses when higher load-carrying capacity is required without resorting to carbon fiber [60]. Glass fibers are extremely versatile and are available in numerous forms to accommodate specific manufacturing processes and component requirements [33]. Chopped strands are applicable in mass production methods such as sheet molding compound (SMC) and bulk molding compound (BMC) for tractor body panels (hoods, fenders, roofs) or via injection molding with thermoplastic matrices for smaller, complex parts. Woven fabrics provide multi-directional reinforcement suitable for hand lay-up or resin transfer molding (RTM) processes to produce tough guards or semi-structural parts [20,53,61,62]. Continuous rovings are required for processes such as filament winding (for high-pressure tanks or pipes) and pultrusion, the latter being used extensively to produce the long, stiff, and light profile sections required for modern sprayer booms [63]. Generally, the cost-effectiveness and well-characterized performance of GFRP have made it the dominant composite reinforcement for a wide variety of agricultural applications where a significant advantage over traditional metals is desired without prohibitive cost increases.
- Carbon fibers: Offering excellent specific stiffness and specific strength—typically three to five times better than that of E-glass on a weight basis—carbon fibers represent the ultimate in lightweight structural reinforcement. Their high fatigue resistance and low density are highly attractive properties [64]. However, their significantly higher cost restricts their use in the typically cost-sensitive agricultural sector to high-value, performance-critical applications [65]. A prime example is in the production of ultra-wide sprayer booms [63]. For such applications, it is desirable to reduce the boom weight to lower soil compaction and increase stability, and to increase stiffness to prevent excessive boom whipping and yawing during operation on uneven terrain. This preserves the precise application height and pattern required for effective and efficient delivery of fertilizers or pesticides, minimizing waste and environmental impact. While GFRP suffices for smaller booms, the superior properties of CFRP become enabling at such wide extremes, justifying the cost premium through higher productivity and accuracy [63,66,67]. Beyond booms, CFRP can find future niche applications in high-speed rotating components where low inertia is most critical or in lightweight structural frames of specialized autonomous farming equipment or drones where minimization of mass is most critical to range or payload capacity.
- Aramid fibers: More commonly referred to by their trade names, e.g., Kevlar^®^, aramid fibers are prized for their toughness, impact resistance, and tensile strength [68]. They possess lower compressive strength compared to carbon or glass fibers and might be susceptible to moisture absorption if not effectively protected by the matrix, which could prove to be a weak point in humid agricultural environments. Due to these limitations, together with their relatively high cost, the use of aramid fibers in agricultural applications remains narrow [69,70,71]. Nevertheless, due to their unique mechanical properties, they represent potential candidates for high puncture or impact resistance parts [72,73]. Potential applications include protective shielding for underbody parts exposed to rock impact (e.g., on tractors or combines operating in rocky fields), durable guards for rotating power take-off (PTO) driveshafts, or reinforcement in specific impact-prone zones on harvesting headers or tillage tools, where localized toughness is more critical than overall structural stiffness [74].

#### 3.1.2. Natural Fibers

**Figure 4:**
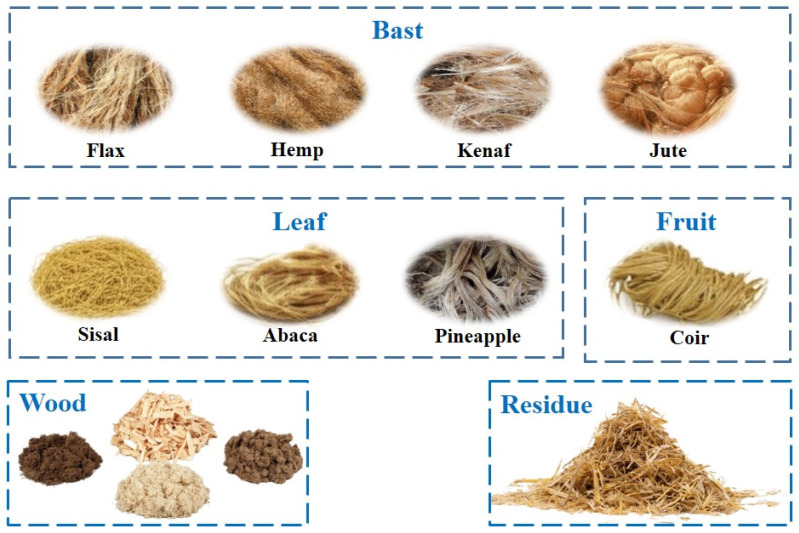
Examples of common natural fibers used in biocomposites (diagram drawn by the authors).

Driven by sustainability goals and the potential utilization of agricultural co-products, natural fibers have garnered increasing interest as reinforcements for biocomposites. Their renewable origin, potential for biodegradability, lower energy requirements for production, and low density are key advantages [16,75]. Among the most used natural fibers in biocomposites are those shown in Figure 4, as an example, to highlight the diversity of these fibers.

- Bast fibers: Extracted from the outer fibrous sheath of the plant stem, bast fibers such as flax, hemp, jute, and kenaf are among the most viable natural reinforcements for semi-structural applications [76]. Flax and hemp, however, possess sufficiently high tensile strength and rigidity, along with specific values of stiffness that are comparable to E-glass, and they are therefore favorable candidates to supplant GFRP in certain applications [77,78]. Their potential uses in agricultural machinery include interior cabin components (dashboards, door panels, headliners, trim pieces), where their look and vibration damping can be beneficial, and non-load-carrying exterior panels such as engine covers, access hatches, or toolboxes [53,79]. Their use in semi-structural elements—such as reinforcing ribs or brackets—is also conceivable, especially when combined with higher-performance thermoplastic or thermoset matrices. Jute is cheaper but typically produces low strength and durability, increased moisture sensitivity, and is likely to be confined to price-sensitive interior filling application or components that are not subjected to significant mechanical loading or exposure to weather [80]. Kenaf offers intermediate properties between jute and flax/hemp and continues to be actively researched [15].
- Leaf fibers: Leaf-derived fibers such as sisal (obtained from Agave species) and abaca (obtained from a banana family member) are documented to have good toughness, durability, and saltwater resistance—properties that have traditionally made them suitable for rope manufacturing [81,82]. While their stiffness is usually less than the best bast fibers, their toughness can be useful in agricultural components with abrasion or localized impact resistance requirements [82]. Prospective applications could include wear liners on material-handling chutes (such as grain or forage), cover plates in less structurally critical areas of tillage or harvest equipment, or elements where a degree of flexibility along with resiliency would be advantageous. However, their use in load-bearing structures is not currently considered as likely as some of the more robust bast fibers such as flax or hemp, but further studies may exhibit new applications.
- Seed/Fruit fibers: Cotton (used primarily in textiles due to its fineness and softness, thus of less interest for structural reinforcement) and coir (from coconut husks) belong to this group [83]. Coir fibers are extremely low in density, highly elongated, possess good resilience, and have relatively good resistance to rot and moisture. However, their mechanical stiffness and strength are comparatively much lower than bast or leaf fibers [84,85]. As such, their role in agricultural machinery composites is unlikely to be in structural reinforcement. They will potentially find other niche applications though as lightweight core material in sandwich panel structures (e.g., platforms or dividers), fillers to keep weight and expense low in components that are non-load-bearing, or possibly within components where damping and energy absorption would be a desirable attribute (e.g., seat padding or sound insulation within tractor cabs) [86,87].
- Wood fibers: Inexpensive, readily available wood industry by-products known as wood fibers are commonly used, particularly in wood–plastic composites (WPCs) [88]. Wood particles are typically blended with thermoplastic matrices such as PE, PP, or PVC, often by extrusion or injection molding [89,90,91]. While WPCs are extremely prevalent in their application for such purposes as outdoor decking, fencing, and window profiles, they are not widely utilized in farm equipment but could provide some opportunities [92,93]. For instance, WPCs can be applied on components such as utility trailer flooring panels or shipping box components, non-load carrying auxiliary equipment coverings, operators’ platforms, or possibly as replaceable wear surfaces where high structural integrity is not a primary requirement and where cost-effectiveness and moderate durability/moisture resistance (relative to untreated wood) are sufficient. However, their mechanical properties are generally inferior to those of continuous fiber-reinforced composites.
- Agricultural residues: Utilization of residues such as wheat straw, rice straw, corn stover (stalks and leaves), or sugarcane bagasse as reinforcement directly falls into circular economy principles in the agricultural sector with the possibility of adding value to huge quantities of low-cost biomass [94]. These are sustainable and abundant materials. Today, a vast quantity of agricultural residues is mostly utilized for energy generation—either as feedstock for biofuels, greenhouse heating, or powering farm operations—rather than being extensively utilized in the production of biocomposites for machinery components [95,96]. Despite such a prevalent trend for energy applications, research continues to seek their utilization as part of composite materials. However, from a mechanical point of view, these residues offer lower strength and stiffness and higher variability compared to dedicated fiber crops such as flax or hemp. Moreover, their harvesting, cleaning, and processing effectively are logistically demanding [97]. Therefore, their current use in agromachinery composites can be primarily as low-cost fillers to reduce material consumption and weight in non-critical components, or as reinforcement in parts subjected to low mechanical loads. Some possibilities include internal panels, sound insulation, or biodegradable components such as temporary plant supports or mulch film holders, where high mechanical strength is not required.

Challenges for natural fibers in agricultural uses remain considerable. Despite the sustainability advantages, the extensive utilization of natural fibers in demanding agricultural equipment uses is faced with great challenges. Their inherently hydrophilic nature leads to excessive moisture absorption in typically damp or humid agricultural environments. This can lead to swelling of the fiber, dimensional instability of the composite, degradation of the fiber–matrix interface, microbial attack, and overall loss of long-term mechanical performance [98]. Additionally, achieving effective interfacial adhesion between polar natural fibers and non-polar polymer matrices remains a key technical challenge [26,99]. Its solution often requires fiber surface treatments (e.g., alkali, silane) or the use of coupling agents, both of which increase the cost and complexity of processing [37,38]. Poor adhesion lowers the ability of the matrix to efficiently transfer stress to the fibers, thereby weakening the structural capability of the composite under mechanical stresses typical of farm machinery [100]. Moreover, the relatively low thermal stability of natural fibers (typically decomposing at above 200–230 °C) limits the selection of available polymer matrices and processing techniques, therefore excluding their use in components exposed to high temperatures—such as areas near engines, exhausts, or hydraulic systems of tractors, combines, and other self-propelled equipment [46]. In recent years, nanofillers such as carbon nanotubes (CNTs) and graphene nanoplatelets (GnPs) have shown promising potential to improve fiber–matrix adhesion. These nanomaterials, when incorporated into the matrix or used as coatings on fiber surfaces, enhance the interfacial bonding through mechanical interlocking, increase the surface area, and improve the stress transfer efficiency [47]. Their addition can mitigate the drawbacks of poor adhesion, leading to composites with superior mechanical integrity suitable for demanding applications in AME.

**Figure 5:**
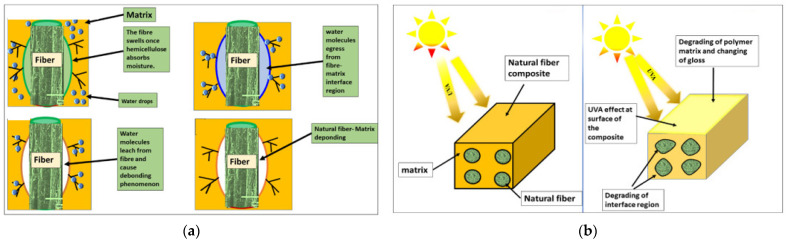
Schematic representation of key degradation mechanisms affecting biocomposites [43]: (**a**) Effect of water on the fiber–matrix interface; (**b**) Deterioration mechanisms caused by high temperatures and ultraviolet light.

As shown in Figure 5, the key degradation mechanisms affecting biocomposites—such as the effects of moisture, temperature, and ultraviolet radiation—are illustrated, highlighting how these environmental factors can compromise the integrity of the fiber–matrix interface and accelerate material deterioration. To expand the reliable application of natural fibers beyond protected, non-structural roles in agriculture, it is crucial to address these limitations through advanced material formulations, durable fiber treatments, optimized matrix selection, and careful component design.

### 3.2. Polymer Matrices

The matrix phase of a composite acts as the continuous phase and plays different important roles: it compacts the reinforcing fibers, provides shape and rigidity to the component, transfers applied loads across the fibers, and protects them from degradation resulting from exposure to the environment (e.g., water, chemicals, UV radiation) and mechanical wear [13,101]. The matrix selection plays a key role in the overall performance of the composite, including its thermal stability, chemical resistance, toughness, processing behavior, and ultimately its performance and durability in the harsh environments experienced in agricultural machinery [51,52,54]. Polymer matrices are broadly classified into conventional thermosets, conventional thermoplastics, and emerging bio-based polymers—each offering distinct advantages and limitations for agricultural applications.

#### 3.2.1. Conventional Thermosets

Thermosetting polymers are treated with irreversible chemical cross-linking during curing that forms a tough three-dimensional network structure. Such a structure confers excellent mechanical strength, heat stability, and chemical resistance but typically renders the material brittle and more difficult to recycle compared to thermoplastics [31,58].

- Unsaturated polyesters (UPs): These are considered the workhorse thermoset resins in many industries, including agriculture, primarily due to their low cost and ease of processing using relatively simple techniques such as hand lay-up, spray-up, and increasingly, compression molding using SMC or BMC [102]. This makes them economically viable for manufacturing large, relatively low-volume components common in agricultural machinery, such as tractor hoods, fenders, roofs, and equipment enclosures. Their good balance of mechanical properties (adequate strength and stiffness for non-primary structural parts), inherent corrosion resistance compared to steel, and ability to be molded into complex shapes are key benefits [102,103]. However, standard UP resins exhibit only moderate chemical resistance, limiting their use in direct contact with aggressive agrochemicals, and their mechanical properties are generally lower than those of vinyl esters or epoxies [104]. Furthermore, processing often involves the emission of volatile organic compounds, such as styrene, raising environmental and workplace safety concerns—issues that are being addressed by newer low-styrene or styrene-free formulations [103,104]. SMC/BMC formulations allow for higher production volumes with better dimensional control and surface finish, making them suitable for series production of panels [102].
- Vinyl esters (VEs): Chemically related to both polyesters and epoxies, VE resins offer significant advantages in enhanced chemical resistance, particularly against hydrolysis, acids, and various solvents commonly found in fertilizers and pesticides [105]. This superior chemical inertness stems from the placement of the reactive ester groups primarily at the ends of the molecular chains, reducing their vulnerability to chemical attack compared to the numerous ester linkages along the backbone of UP resins. Consequently, VE resins are the preferred matrix material for manufacturing durable chemical storage tanks and sprayer tanks in agricultural equipment, ensuring containment integrity, preventing the contamination of tank contents, and prolonging service life in chemically aggressive environments [106,107]. While more expensive than UP, their enhanced performance justifies the cost for these critical applications. VE resins also generally exhibit better mechanical properties (e.g., strength, toughness) than UP and can be processed using similar methods including hand lay-up, resin transfer molding, and filament winding for tanks [106].
- Epoxies (EPs): Epoxy resins are renowned for their superior mechanical properties (high strength, stiffness, and fatigue resistance), excellent adhesion to a wide range of fibers, good dimensional stability, and broad chemical resistance [106,107]. This performance profile makes them the matrix of choice for high-performance composite applications in agriculture. Their excellent adhesion is critical for maximizing the performance of expensive reinforcing fibers such as carbon, hence their prevalent use in CFRP components including ultra-wide sprayer booms, where efficient stress transfer ensures the required stiffness and strength are obtained at minimum weight [63,108]. Epoxies may also be used with glass fibers for components subjected to higher structural loads, significant fatigue cycles, or demanding durability requirements—beyond what is achievable with UP or VE—such as load-bearing brackets or reinforcement structures [63,109]. However, epoxies are typically more expensive than polyesters or vinyl esters and often require longer, more carefully controlled curing cycles, sometimes at elevated temperatures, increasing manufacturing complexity and cost. Their superior performance comes at a premium, limiting their widespread use to applications where the performance benefits outweigh the additional expense [106,107].

#### 3.2.2. Conventional Thermoplastics

Thermoplastic polymers soften upon heating and solidify upon cooling, a reversible process that enables reshaping, reforming, and efficient recycling. Compared to thermosets, they generally offer superior toughness and impact resistance. Additionally, processing methods such as injection molding facilitate the high production rates of complex-shaped components [80,101].

- Polypropylene (PP): As one of the most common thermoplastics utilized, PP offers an advantageous combination of low cost, inertness to bases and acids (especially), and low density (~0.9 g/cm^3^) with good processability, particularly through injection molding [110]. In agriculture applications, PP is employed similarly to its use in the automotive sector—for components such as interior parts of tractor cabs (e.g., dashboards, trim panels, and consoles), enclosures for auxiliary equipment, protective covers, and small tanks or containers for non-aggressive liquids [53,111]. When reinforced with short or long glass fibers, its mechanical properties—strength, stiffness, and creep resistance—are significantly enhanced, making it suitable for semi-structural components [66,67]. PP is also widely used as a matrix for natural fiber composites (e.g., flax/PP, hemp/PP) and wood–plastic composites, offering a viable pathway for integrating renewable materials into cost-sensitive applications [112]. However, limitations include its relatively low service temperature (compared to engineering thermoplastics such as PA) and moderate UV resistance, which necessitate stabilization for prolonged outdoor exposure [110].
- Polyamides (PAs, Nylons): Thermoplastics such as PA6 and PA66 possess higher strength, stiffness, wear resistance, and thermal properties than PP [113]. These materials can be used in combination with glass fibers (GF/PP) to produce more demanding agricultural components, including moderately loaded gears (when lubricated), wear pads, robust enclosures located near heat sources, fan blades, and structural brackets that require a higher load-bearing capacity than those made with GF/PP [114,115,116]. However, polyamides are more costly than PP and exhibit a notable drawback in agricultural applications—moisture absorption [113]. In the typically damp environments of farming, absorbed moisture acts as a plasticizer, reducing the stiffness and strength of the material and leading to dimensional changes that must be considered during component design.
- Polyethylene (PE): Available in various densities (e.g., LDPE, HDPE), PE is valued for its excellent chemical resistance, impact toughness (especially in HDPE at low temperatures), low cost, and ease of processing [34]. High-density polyethylene (HDPE) is widely used in rotational molding to produce large, seamless tanks for storing water, liquid feed, diesel fuel, diesel, or non-aggressive chemicals, offering a cost-effective and durable alternative to GFRPs [117,118]. Due to its chemical inertness, PE is highly suitable for such containment applications. It is also commonly used as a matrix in extruded WPC profiles for non-structural parts [88]. However, PE’s lower strength, stiffness, and thermal resistance, compared to PP or PA, limit its suitability for load-bearing or high-temperature machinery components.

#### 3.2.3. Bio-Based Matrices

Driven by sustainability imperatives, bio-based polymers derived from renewable resources (e.g., plants, microorganisms) have emerged as alternatives to conventional petroleum-based matrices. These materials aim to reduce the reliance on fossil fuels, lower carbon footprints, and potentially offer biodegradability as an end-of-life solution [34,41].

- Polylactic acid (PLA): Derived from fermented plant starch (e.g., corn, sugarcane), PLA is among the most commercially available bio-based compostable polymers. It exhibits good stiffness (comparable to polystyrene) and transparency, making it suitable for certain non-load-bearing applications [51,76]. However, its use in durable agricultural machinery is limited by its inherent brittleness, low heat distortion temperature (typically around 50–60 °C), and susceptibility to hydrolysis—factors that impair its performance in hot or humid agricultural environments [119]. As a result, PLA is more appropriate for short-term items (e.g., biodegradable clips, tags, or planting aids), protected interior components, or packaging. Ongoing research focuses on enhancing PLA’s toughness and thermal resistance through blending, copolymerization, or fiber reinforcement [120].
- Polyhydroxyalkanoates (PHAs): These are polyesters biosynthesized by various microorganisms [121]. A key advantage of PHAs is their biodegradability in a wide range of environments, including soil and aquatic systems—unlike PLA, which requires industrial composting. The properties of PHAs can be tuned from rubbery to stiff by modifying the fermentation process and bacterial strains [122]. Nevertheless, PHAs are currently limited by high production costs and thermal instability during processing. Consequently, their use in agricultural machinery is confined to niche applications where biodegradability in soil is a critical requirement (e.g., coatings for slow-release fertilizers, or biodegradable sensor housings) [123,124].
- Bio-based Polyethylene (Bio-PE): Produced from ethanol derived from sugarcane fermentation, Bio-PE is chemically identical to fossil-based PE [125]. Such “drop-in” compatibility allows it to be directly substituted for traditional PE in existing applications—such as rotationally molded tanks or WPC production—without sacrificing performance or the need for equipment redesign. Its main benefit is the use of renewable feedstocks, thereby reducing the carbon footprint.
- Other promising bio-based options: High-performance bio-based matrices are gaining attention. Bio-based polyamides (e.g., PA11 derived from castor oil) have the same characteristics as conventional PAs, including chemical and heat resistance, and might therefore find application in components such as fuel lines and hydraulic systems [126]. Likewise, bio-based polyesters such as polybutylene succinate (PBS) and polytrimethylene terephthalate (PTT) offer varying mechanical properties and degrees of biodegradability [127]. Vegetable oil-, lignin-, or other biomass-based bio-derived epoxies and polyurethanes are also being created as sustainable alternatives to thermosets in structural composites [128,129]. Despite their potential, the broader application of these new bio-matrices in agriculture will depend on overcoming challenges related to cost competitiveness, large-scale manufacturability, long-term durability, and effective end-of-life strategies.

### 3.3. Fillers

Along with strengthening fibers and the binding matrix, fillers are a third important class of constituents routinely added to composite materials. Along with traditional applications such as cost-reducing agents by substituting more expensive polymer resin, fillers are now increasingly selected for their ability to add a specific functional advantage to the composite, e.g., higher stiffness, dimensional stability (reduced shrinkage and warpage), hardness, wear resistance, thermal performance (thermal conductivity or insulation), changed electrical properties, or improved fire retardancy [113,125].

The impact of fillers on the final properties of the composite is complex and is a function of numerous parameters: type of filler (inorganic or organic/bio-based), particle size and form (aspect ratio), volume fraction, dispersion quality in the matrix, and interfacial adhesion between filler particles and the polymer that surrounds them [130]. Proper filler selection requires the assessment of wanted property enhancement against potential trade-offs, especially on the mechanical strength, impact resistance, and component weight—important factors in AME applications.

#### 3.3.1. Mineral Fillers

These inorganic materials are widely used due to their availability, low cost, and ability to impart specific improvements in mechanical and thermal performance.

- Calcium carbonate (CaCO_3_): A very ubiquitous and inexpensive mineral filler, precipitated or ground CaCO_3_ is usually added to thermoset (e.g., SMC/BMC) and thermoplastic composites to reduce costs. It is accountable for raising the stiffness and hardness of the composite [131]. Its relatively high density (approximately 2.7 g/cm^3^) makes it so that it can contribute to the composite density, potentially negating weight-saving objectives gained from fiber reinforcement [132]. This compromise can be acceptable in applications where lightness is less critical, such as immovable housing or ballast components. However, overloading reduces impact resistance due to agglomeration or poor interfacial adhesion, which creates stress concentration points [131,133]. Therefore, its use in impact-sensitive parts such as bumpers needs to be carefully formulated.

**Figure 6:**
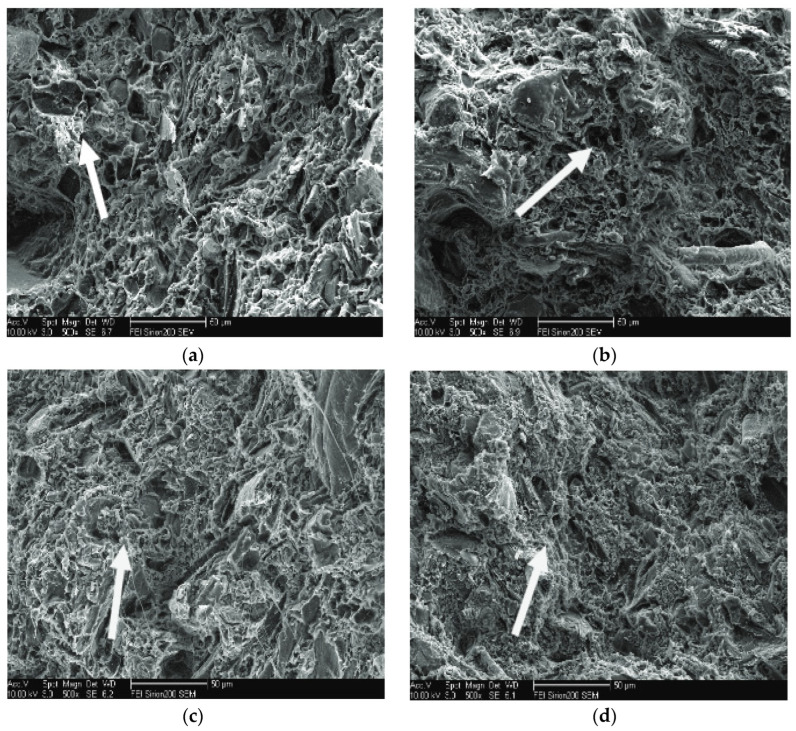
SEM micrographs of WPC fracture surfaces under different calcium carbonate contents [133]: (**a**) Without calcium carbonate (×500); (**b**) Added 5% calcium carbonate (×500); (**c**) Added 10% calcium carbonate (×500); (**d**) Added 30% calcium carbonate (×500).

Based on the SEM images presented in Figure 6, the effect of calcium carbonate addition on the fracture morphology of WPCs has a direct bearing on their application in AME parts. In the untreated WPC sample (Figure 6a), the presence of extensive fiber pull-out and a rough fracture surface indicates poor interfacial adhesion, which can lead to premature failure when subjected to mechanical loading in agricultural use, i.e., in panels or housings exposed to impact or vibration. By incorporating calcium carbonate (Figure 6b,c), particularly up to 10%, the microstructure becomes denser and more cohesive with reduced fiber pull-out and improved matrix-filler adhesion. The implication is higher energy absorption and toughness—properties critical for exterior guards, floors, or internal cabin components in tractors and harvesters that are subjected regularly to shock loading. However, at a high filler content (30% in Figure 6d), indications of filler agglomeration and microvoids are more pronounced. Such features may act as initiation sites for cracks, reducing the resistance of the material to cyclic or impact loading prevalent in agricultural applications. Therefore, optimizing the calcium carbonate content is essential to improve the mechanical performance and reliability of composite materials used in non-structural yet load-sensitive agricultural machinery parts.

- Talc: A hydrated magnesium silicate with a platy (lamellar) structure, talc provides notable stiffness and flexural modulus improvements, often outperforming particulate fillers at similar loadings [134]. Talc also enhances dimensional stability and warpage minimization, beneficial for producing precise parts such as dashboards or ventilation components. Furthermore, talc increases the heat deflection temperature (HDT), making it a good choice for employment in the vicinity of engines or hydraulic systems [135].
- Silica (SiO_2_): Silica, available in various forms such as ground quartz, fumed silica, and precipitated silica, primarily enhances the composite’s hardness, scratch resistance, and compressive strength [136]. Due to its abrasive nature, it significantly enhances wear resistance—a very important property for agricultural machinery exposed to friction or contact with soil, crops, or granular materials. It is also used as a filler in expert wear-resistant coatings applied to tillage machines or harvester parts [137]. Although these coatings are metallic or ceramic, polymer composites filled with silica can serve as an economical replacement for moderate wear applications. Moreover, fumed or precipitated silicas, due to their extremely small particle size and high surface area, find applications in dilute concentrations as rheology modifiers (thixotropes) in liquid thermoset resins such as polyesters and epoxies [138,139]. These additives are utilized for regulating resin flow during processing, inhibiting drainage on vertical surfaces when the lay-up of large tractor body panels is manual, and imparting a uniform thickness throughout the composite.
- Other mineral fillers: Nanoclays (e.g., montmorillonite) enhance barrier properties, stiffness, and flame retardancy at low loadings [140]. Mica improves the stiffness and insulation properties [141]. Glass microspheres reduce the density while maintaining stiffness, making them suitable for lightweight parts such as core materials or molded housings [142].

#### 3.3.2. Bio-Fillers

Bio-fillers such as wood flour, rice husk ash (RHA), straw/stover powder, and diatomaceous earth (DE) in the field of agricultural machinery and equipment provide promising alternatives in the development of composite and biocomposite materials. These fillers are primarily derived from agricultural and forest residues and are compatible with sustainability goals due to their low cost, low density, renewability, and biodegradability. Wood flour and straw powder primarily function as cost-reducing extenders with minimal reinforcement effect and are highly sensitive to moisture, which limits their use in high-durability applications [143]. RHA, rich in silica, can enhance hardness and wear resistance, making it suitable for components exposed to abrasion, although it may cause wear on processing equipment [144]. DE, with its light and porous framework, offers benefits such as increased stiffness, impact resistance, and heat insulation—useful for parts such as tractor cabs or protective coverings [145]. While beneficial, bio-fillers are plagued by disadvantages such as the excessive absorption of water, poor dispersion in polymer matrices, poor thermal stability, and poor interfacial adhesion with hydrophobic polymers. These limitations necessitate careful matrix choice, the use of compatibilizers, and surface treatment to ensure adequate stress transfer and long-term durability in agricultural applications.

## 4. Fabrication Processes of Composites and Biocomposites

The selection of a suitable manufacturing process is one of the determinants of composite and biocomposite part performance, quality, and cost for agricultural machinery and equipment. Low-cost, manual methods such as hand lay-up and spray-up are suitable for prototype and big, simple parts but are riddled with inconsistent quality and poor mechanical properties due to voids and low fiber content fractions [24,31,77,102]. More expensive closed-mold techniques such as compression molding and RTM/VARTM offer better mechanical properties, surface finish, and reduced emissions and are thus better suited for the medium- to high-rate production of structurally demanding AME parts [53,58,91]. Pultrusion and injection molding are best suited for the high-volume, high-efficiency production of constant-profile and complex-geometry parts, respectively, although both have design and material limitations [33,59,97,112]. Extrusion is being used routinely in continuous thermoplastic profiles and in WPCs for non-structural AME parts [28,37,141].

**Figure 7:**
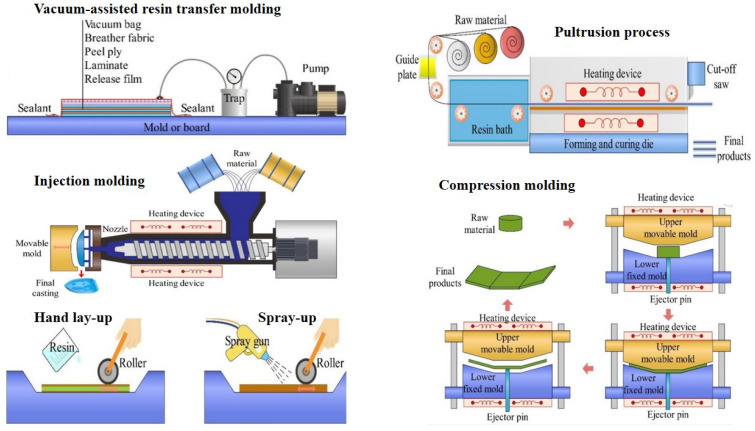
Schematic representations of common composite fabrication methods used in agricultural machinery and equipment applications [45].

For biocomposites, natural fibers’ heat and water sensitivity, along with the processing restrictions of fiber degradation and wet-out, must be dealt with tactfully in order to deliver structural integrity within stress-requiring agricultural conditions. These processes and their configurations are schematically illustrated in Figure 7.

## 5. Applications of Composites and Biocomposites in Agricultural Machinery and Equipment

The use of composite materials, especially GFRP, in agricultural machinery and equipment has increased due to their lightweight nature, corrosion resistance, and design flexibility. These advantages contribute to improved fuel efficiency, reduced soil compaction, and longer component life in harsh agricultural environments [62,66]. Although GFRP is predominantly used, high-performance CFRP is used in demanding applications, and biocomposites are also being researched more and more for the environmental benefits in less structurally demanding components [20].

### 5.1. Tractor Components

- Exterior panels (hoods, fenders, roofs): GFRP is suitable for use in exterior panels, offering 25–40% weight savings compared to steel. These parts resist corrosion and impacts and allow for modern aerodynamic designs [62,66]. Biocomposites such as hemp/PP and flax/PP can also be used for these non-structural components, especially where sustainability is prioritized [3,112]. Findings from the automotive sector regarding non-structural biocomposite panels are often directly transferable, although long-term UV stability and impact resistance in the harsher AME environment require specific validation.
- Interior cab parts (dashboards, trim panels): Thermoplastic composites reinforced with glass or natural fibers (e.g., flax/PP, WPCs) are used for their weight reduction, integrated design features, and acoustic insulation, enhancing operator comfort.

### 5.2. Sprayer Components

- Tanks: Holding potentially corrosive liquid fertilizers, pesticides, and herbicides requires materials with excellent chemical resistance. GFRP, particularly when fabricated using specialized chemical-resistant VE or isophthalic polyester resins, is considered the industry standard for sprayer tanks [106,107]. These materials eliminate the risk of rust contamination associated with steel tanks, ensuring chemical purity and preventing nozzle blockages. Their lightweight nature allows manufacturers to either increase tank capacities—thereby improving field efficiency by reducing refill stops—or reduce the overall vehicle weight, which contributes to lower soil compaction and enhanced maneuverability, especially on softer terrain. For smaller-sized tanks or sprayers used to contain less-aggressive chemicals, rotationally molded PE tanks also offer an economic, seamless, and chemically resistant option.
- Booms: The large truss booms carrying spray nozzles are one of the most conspicuous and impactful applications of high-performance composites in AME. As boom widths increase (often exceeding 30 m) to enhance productivity, the weight and inertia of traditional steel or aluminum booms become significant limitations, leading to instability, increased soil compaction under the wheels, and potential structural fatigue [146]. GFRP booms, typically manufactured using pultrusion for the main profiles or resin transfer molding for sections and joints, offer substantial weight savings—often a 30–50% reduction compared to steel [63]. This weight reduction improves boom stability, allowing for a more consistent spray height and application patterns, which is critical to spray efficacy and reducing drift. For extremely wide booms (over 36–40 m), the greater specific stiffness of CFRP becomes the decisive factor [63]. CFRP allows these ultra-wide structures to maintain sufficient rigidity, preventing excessive deflection and vibration, thereby ensuring precise spray application, which is vital for precision agriculture practices. Although CFRP is much more expensive than GFRP, cost justification is provided by the performance enhancement—including the higher work rate, reduced chemical usage through more precise targeting, and lower field passes that minimize compaction—where the expenditure on CFRP is justified on premium class large-scale spraying operations.

### 5.3. Combine Harvester Components

- External panels and covers: Similar to tractors, heavy external panels (e.g., side shields, engine covers), protective guards, and access doors are typically capable of being manufactured from GFRP through techniques such as sheet molding compound or lay-up processes. Not only does this make the machine lighter in general but also provides high resistance to corrosion from plant juices and moisture. Additionally, GFRP offers enhanced durability against impacts from crop stalks or field debris, ensuring longer service life and improved performance in harsh agricultural environments. In addition to GFRP, biocomposites (e.g., bamboo/flax) are also applicable to the design and manufacture of exterior and protective components. As seen in recent years, these materials have been successfully employed in the automotive industry, providing an eco-friendly alternative with similar benefits, such as weight reduction and improved performance [147].
- Internal components: While metals continue to dominate most internal components due to their high abrasion resistance to grain and crop materials, there is growing interest in the use of composites for some internal components. Some potential applications are grain handling parts, such as auger troughs and elevator housings, where the lighter weight would decrease drive power requirements. Abrasion resistance, however, remains a key issue and may require special wear-resistant coatings or composite structures. Components used in the separation of grain from chaff, such as shaker pans or sieve frames, could benefit from the reduced inertial loads of lightweight composites. This could allow for increased operating frequencies or reduced transmission of vibration, leading to improved efficiency. However, the widespread adoption of composites in these applications is hindered by concerns about their durability under continuous vibration and impact from grain, as well as cost considerations. Biocomposites will find limited use in non-load bearing, non-abrasive internal covers or ducting, where their environmental benefits align with performance requirements.

### 5.4. Planting and Seeding Equipment

- Hoppers: Fertilizer and seed hoppers may be made from composites such as GFRP or specially formulated biocomposites. The most significant advantage of using such a material is the excellent resistance to corrosion from hygroscopic and potentially acidic fertilizers. The composites also enable the production of smooth, molded shapes that facilitate consistent material flow towards the metering system, thereby preventing bridging or clogging and ensuring accurate application rates. Their lightweight characteristics contribute to easier handling and lower draft force requirements during operation. It is also feasible for certain GFRP hoppers to be designed to be transparent, so the material level can be seen by operators without the necessity of additional sensors.

### 5.5. Other Equipment and Components

Various agricultural machinery components benefit from the use of composite materials due to their advantageous properties. In fertilizer spreaders, GFRP can be used for hoppers and bodies due to its excellent resistance to corrosion caused by aggressive chemical salts. Protective housing for electronic control units, sensors, and wiring harnesses may be manufactured from GFRP or injection-molded thermoplastics, providing environmental protection, impact resistance, and electrical insulation. Additionally, safety guards around rotating shafts, belts, and chains may be manufactured from composites to enhance durability while reducing weight. Specialized harvesting equipment for delicate crops such as fruits and vegetables may integrate composites into parts including robotic arms, picking fingers, or conveyors—where lightweight structure, controlled flexibility, and impact resistance are essential for efficient operation and crop protection.

Thus, the use of composite materials—particularly GFRP—is well-established across various AME components, where their advantages in lightweighting, corrosion resistance, and design flexibility offer clear performance and durability benefits over traditional metals. CFRP also plays a crucial role in enabling high-performance systems, such as ultra-wide sprayer booms, by providing superior stiffness-to-weight ratios. While biocomposites present strong sustainability potential, their current application remains largely limited to non-structural or less-demanding parts, primarily due to challenges related to cost and long-term durability under harsh field conditions. Nevertheless, continued advancements in composite materials and manufacturing technologies are expected to drive their broader adoption in AME, aligning with industry goals to improve operational efficiency, reduce environmental impact, and enhance the overall productivity and sustainability of modern agricultural practices.

### 5.6. Review of Existing Research and Technological Trends

**Figure 8:**
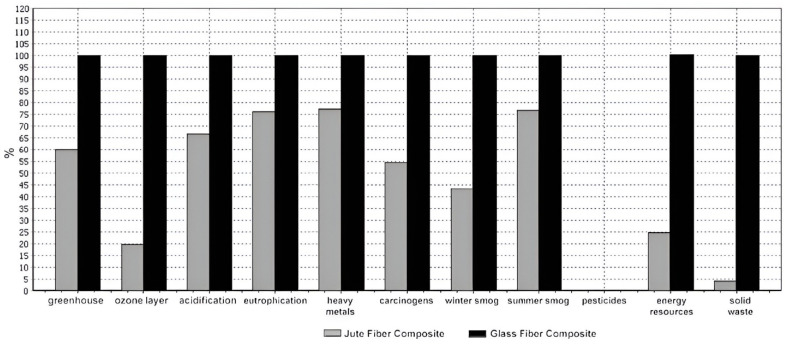
Comparison of sustainability scores of materials used in agricultural machinery components based on the sustainable design procedure, with permission from Elsevier [20].

The increasing demand for sustainable solutions in AME has led to the exploration of composite and biocomposite materials as alternatives to traditional metals. Alves et al. (2009) in this case proposed a sustainable design procedure aimed at integrating environmental considerations into material selection for AME components, particularly focusing on self-propelled sprayer machines [20]. A key contribution of the study lies in the comparative assessment of traditional GFRP and natural fiber composites (e.g., jute, sisal, coir) using weighted performance indices that account for mechanical, aesthetic, processing, and environmental parameters. Notably, jute fiber composites demonstrated comparable tensile performance to GFRP in both physical testing and finite element analysis (FEA) while offering significant environmental advantages, especially in terms of reduced energy consumption, material renewability, and lower greenhouse gas emissions during the use phase (Figure 8). The study also presented a life cycle assessment to highlight the environmental benefits of natural fiber composites beyond the manufacturing process. The results revealed that jute composites outperformed GFRP in several environmental impact categories (e.g., CO_2_ emissions, acidification, eutrophication), particularly when accounting for fuel savings from reduced agricultural machine weight (Figure 8). These findings reinforce the suitability of vegetable fiber-reinforced composites as promising eco-efficient alternatives in AME, especially for components where lightweighting and sustainability are critical.

**Figure 9:**
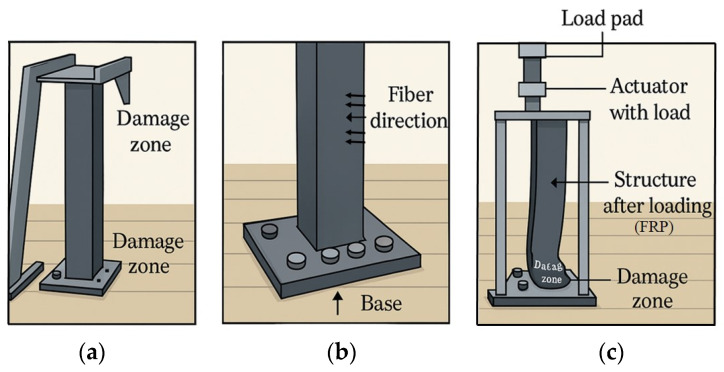
Development, testing, and failure sequence of the FRP ROPS: (**a**) Development apparatus; (**b**) Base fixture before loading; (**c**) Structure (FRP) after loading to failure (authors’ own drawings).

Etherton et al. (2007) investigated the application of pultruded GFRP in the production of rollover protective structures (ROPSs) for agricultural tractors [148]. The motivation stemmed from the need to reduce tractor rollover fatalities in agriculture—a major cause of occupational deaths—by offering lighter and more cost-effective protective structures that are easier to deploy than traditional steel-based ROPSs. Notably, their laboratory tests demonstrated that well-designed bolted joints and cantilevered GFRP members could meet or exceed the strength requirements of steel ROPSs defined by the SAE J2194 standard [149]. To aid in achieving a better understanding, overall, the study laid a foundational understanding for the integration of composite materials in agricultural safety structures, highlighting the potential of GFRP and similar materials to enhance safety, reduce tractor weight, and lower production costs in the AME sector. Figure 9 provides a schematic illustration of the appropriate positioning of FRP elements within the ROPS, along with a representation of the loading tests conducted on the FRP elements.

Misirli et al. (2014) studied the use of GFRP composites for tractor bonnets with vinyl ester resin and orthophthalic resin as matrices with chopped, felt, and woven glass fiber reinforcement [150]. The outcome showed that there was superior heat behavior of the vinyl ester resin with a higher glass transition temperature (T_g_) at 109.6 °C compared to the 91.9 °C of orthophthalic resin, making it more appropriate for use under variable agricultural conditions. SEM analysis of the fracture surfaces revealed improved fiber orientation and uniform crack propagation in the vinyl ester composites. Mechanical testing also confirmed that vinyl ester composites with four layers of woven glass fiber exhibited the best tensile strength (283.4 MPa) and flexural strength (523.9 MPa).

Mlýnek et al. (2019) proposed a novel technique for composite frame design in agricultural machinery based on automated fiber winding with the help of an industrial robot [62]. Their method was based on determining the optimal off-line motion of the robot-end-effector to achieve precise fiber placement on the composite frame, enhancing its mechanical properties and end-structure uniformity. The authors employed a mathematical model incorporating matrix calculus to guide the frame through a fiber-processing head in a manner that ensures correct winding angles and homogeneity. This automated approach not only minimized manual handling but also allows us to produce lighter, corrosion-resistant, and structurally optimized frames for tractors and other agricultural equipment—ensuring opportunities for more complex and durable composite structures in the AME sector.

Mlýnek et al. (2022), advancing their prior research, refine the fiber winding process for composite frames with varying cross-sectional radii and load-specific winding angles, optimized using ANSYS and ABAQUS software [151]. The study introduces mathematical equations to control the angular velocity and winding distance to enable the precise placement of fibers on multi-component frames. This process assists in the production of light, fuel-efficient agricultural machinery with enhanced durability that suits differential load requirements and assists in minimizing environmental stresses such as soil compaction through lighter machine weights.

The development of advanced polymer composites for AME has gained attention for enhancing performance and durability. Vodyakov et al. (2019) investigated the modification of PA6 with 2.5% shungite and graphite fillers to improve its mechanical and tribological properties for hydraulic cylinder components [152]. Their findings revealed a 25–27% increase in tensile strength, an 85% boost in elastic modulus, and a 23% reduction in the friction coefficient compared to unmodified PA6, alongside a 30–33% decrease in wear intensity under dry and lubricated conditions. The use of a HAAKE PolyLab Rheomix mixer ensured good dispersion, producing a spatial network of physical bonds. Additionally, the composite recorded an 82% cost reduction compared to the commercial UPA6-15A, demonstrating its potential for cost-effective, high-performance applications in AME.

Technological advancements in potato harvesting have also concentrated on reducing energy consumption and improving operational efficiency using novel materials. Zhbanov et al. (2020) investigated the potential of composite materials in harvester components by replacing traditional metal rods in the separating elevator with composite rods [153]. The results showed significant mass reduction of around 50%—from 38.1 kg to 24.2 kg—with a specific energy consumption reduction from 0.54 kW/t to 0.42 kW/t. Additionally, the separation efficiency improved by 10–15%, while tuber damage was reduced by 9–12%. Rubberized shakers with flexible composite rods were effective in reducing shock loads, and their separation rates varied between 86.1% and 92.2%. This modernization enhanced the energy efficiency and mechanical reliability, bringing to the forefront the use of composite materials to maximize agricultural machinery performance.

**Figure 10:**
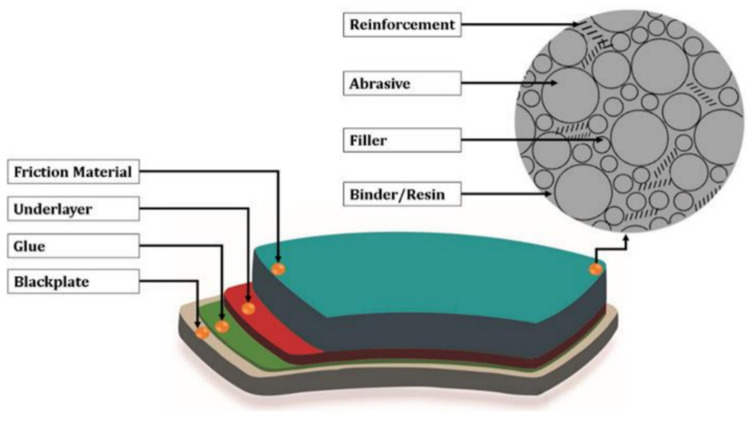
Schematic diagram of brake pad layers and friction material composition [155].

Zagurskiy (2024) examined the development of brake pads for mobile agricultural machinery, focusing on material selection and tribological performance [154]. The study categorized brake pads by purpose, design, friction material (semi-metallic, non-asbestos organic, ceramic), and sensor integration. Modern brake linings were described as composites formed by hot pressing, composed of phenolic resin binders, structural fibers (metal, carbon, glass, Kevlar), fillers (e.g., mica, vermiculite), and friction modifiers (e.g., graphite, metal sulfides). The layered structure of brake pads is shown in Figure 10. Ceramic materials provided stable friction and high heat resistance, while natural fibers such as rice husks enhanced environmental sustainability. The study emphasizes the need for durable, heat-resistant, and eco-friendly composites in agricultural braking systems.

**Figure 11:**
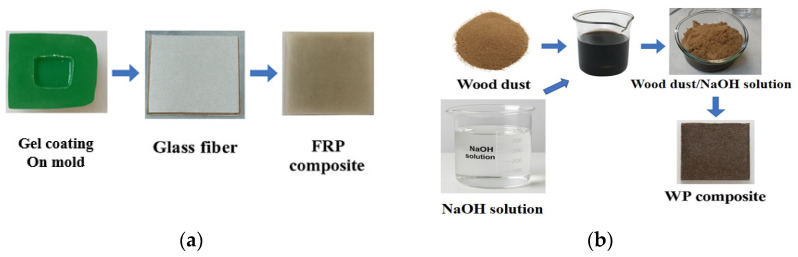
Fabrication processes of (**a**) FRP; (**b**) WP composites, adapted from [24].

Patel et al. (2025) compared WPCs and FRPs for agricultural equipment applications [24]. They found that WPCs and thermoplastics such as PE or PVC provided cost-effectiveness, biodegradability, and moderate strength, ideal for non-load-bearing parts including panels and tool handles. In contrast, FRPs, with glass or carbon fibers in a polymer matrix, offered superior strength-to-weight ratios, corrosion resistance, and durability, suited for structural components such as fenders and tanks. WPCs promoted sustainability through renewable sourcing, while FRPs excelled in harsh environments, minimizing maintenance. The study proposed a hybrid approach combining WPCs and FRPs to optimize performance, cost, and environmental impact in farm machinery design. Figure 11 illustrates a representative fabrication process for both types of composites.

## 6. Advantages and Challenges

The integration of composite and biocomposite materials into the design and manufacture of AME is a delicate balance of great opportunities and great challenges. Although these new materials offer robust solutions to most of the limitations of traditional metals, their effective application entails careful consideration of their unique properties, performance demands, manufacturing requirements, cost considerations, and long-term behavior in the specific, often harsh, field conditions of agriculture.

A general overview of the material properties of interest to agricultural machinery and equipment is presented in Table 1, and the advantages and limitations of such materials are elaborated in detail in the following sections.

### 6.1. Advantages

The application of composites and biocomposites in AME is driven by several intrinsic advantages over conventional metallic materials.

#### 6.1.1. Lightweighting

One of the most significant reasons for the utilization of composite materials in AME is their great ability to reduce weight. Traditional metals such as steel (~7.8 g/cm^3^) and aluminum (~2.7 g/cm^3^) add significant weight to machinery. In contrast, conventional composites including GFRP (~1.8–2.1 g/cm^3^) and CFRP (~1.5–1.6 g/cm^3^), and more specifically biocomposites and natural fiber-reinforced polymers (NFRPs, ~1.1–1.4 g/cm^3^), yield substantially lower densities with corresponding considerable weight reductions (Table 1).

Weight reduction has major performance benefits. Lighter machines cause less soil compaction, preserving soil structure, enhancing aeration and water infiltration, and supporting better crop yields—particularly important in wet or sensitive field conditions [165]. Lower mass also decreases propulsion and operating energy needs, making it more fuel efficient, lowering operating expenses, and facilitating greenhouse gas emission reduction.

#### 6.1.2. High Specific Strength and Stiffness

In mechanical properties, steel possesses high absolute strength and stiffness but low specific strength and stiffness (performance per unit weight) because of its high density. Aluminum possesses moderate specific properties, as shown in Table 1. Conventional FRPs possess high specific strength and stiffness, and GFRP is a highly strong, cost-effective substitute for metals for most structural and semi-structural components. CFRP possesses extremely high specific strength and stiffness, and it is the material of choice for high-performance applications where significant weight savings are required, such as long sprayer booms, despite its much higher cost.

NFRPs generally offer low to medium specific strength and medium specific stiffness. Even though their specific stiffness can be as high as that of GFRP, their strength tends to be lower, and both can be very variable. The variability is based on factors such as fiber type, processing method, fiber content, and quality of the fiber–matrix interface. Biocomposites may also inherit the mechanical limitation of their bio-matrix (e.g., lower strength or stiffness in PLA compared to epoxy or polypropylene), further limiting their application in load-bearing structures.

#### 6.1.3. Corrosion and Chemical Resistance

Durability in the harsh agricultural environment is critical. Unlike steel, which is very susceptible to rust, polymer matrix composites are naturally resistant to electrochemical corrosion [166]. This makes them highly useful to AME components that are consistently exposed to rain, humidity, contact with soil, fertilizers (typically corrosive salts such as nitrates, phosphates, potassium chloride), pesticides, herbicides, and manure [8,20]. FRPs generally exhibit good to excellent chemical resistance, especially if the proper resins, e.g., vinyl esters, are used for applications such as chemical tanks (Table 1) [106,107].

The chemical resistance of NFRPs and biocomposites is largely characterized by the employed polymer matrix—e.g., PP is resistant, but PLA is more hydrolytically susceptible [110]. This resistance produces a longer lifespan for parts, reduces degradation, and optimizes part service life such as tanks, hoppers, spreader bodies, fender liners, and weather- or chemical-exposed structural parts. Additionally, it prevents the need for constant repainting or applying protective coating required with steel; thus, the cost of ownership throughout the lifespan is reduced. Moreover, it improves efficiency by precluding rust contamination in tanks and hoppers, keeping chemicals applied clean, and reducing the risk of clogging in seed meters or sprayer nozzles.

#### 6.1.4. Design Flexibility

Composite fabrication techniques, particularly molding, permit the creation of complex, three-dimensional shapes that are often problematic or costly to achieve using metal forming or casting [167].

The design flexibility confers several advantages: Part consolidation, for instance, enables multiple metallic components to be integrated into a single molded composite component, reducing the number of fasteners, assembly processes, and potential areas of failure or corrosion. Additionally, complex curvatures provide opportunities for aerodynamic and aesthetic styling, improving airflow for cooling or reducing drag while also enhancing the visual appeal of machinery. Lastly, functional optimization allows shapes to be tailored for specific purposes, such as designing smooth, easy-to-clean interiors for tanks and hoppers to avoid residue accumulation or integrating mounting bosses and channels into structural components to optimize performance and convenience.

#### 6.1.5. Sustainability Potential

Metals are highly recyclable and thus a sustainable choice in most uses. However, thermoset composites such as GFRPs and CFRPs present significant recycling challenges (Table 1). These materials are typically non-reprocessable and often end up in landfills or require energy-intensive recycling methods, such as pyrolysis [164,168]. On the other hand, thermoplastic composites, including many NFRPs and biocomposites, offer improved recyclability through processes such as melting and remolding. Regarding manufacturing impact, metals and CFRPs are associated with high energy consumption, while GFRP production is considered moderately energy intensive [159,169]. Natural fiber production generally consumes less energy than glass fiber production, potentially leading to a smaller environmental footprint for biocomposites [38,159]. Nevertheless, comprehensive life cycle assessment studies are essential to fully evaluate and compare the sustainability profiles of these materials.

#### 6.1.6. Other Advantages

Composites naturally exhibit better thermal and electrical insulation [170]. The fiber structure and polymer matrix also provide sufficient damping of vibration, which can be supplemented by the cellular composition of natural fibers [171]. This characteristic reduces noise and vibration for operator comfort in cabs. In addition, it can help extend the lifespan of sensitive electronics equipment by protecting it against aggressive vibrations [41,42].

### 6.2. Challenges

#### 6.2.1. Costs

Cost remains a significant barrier to the widespread application of composite materials, especially when compared to traditional steel. While GFRP material prices have decreased in recent years, it remains more expensive per kilogram than steel, while CFRPs are significantly more expensive due to their high-performance characteristics. As such, CFRPs tend to be utilized where performance enhancements warrant the extra expense [169]. Biocomposites have a more complex cost situation; although raw natural fibers are inexpensive, the need for fiber treatments, quality control to reduce natural variability, special bio-based resins, and perhaps lower process speeds can drive the overall cost to the same or even greater levels than GFRP [31].

The manufacturing costs also contribute to the high overall cost. Resin transfer molding, compression molding, and injection molding are processes that require high up-front capital investment in equipment and tooling. These high up-front costs must be amortized over high volumes of production, which may be challenging for low-volume applications in the AME sector. Despite these higher initial costs, composites may offer cost advantages over their entire operating life. Reduced maintenance, extended service life, and fuel savings through lighter-weight components make composites cost-effective on a life cycle basis, even though these benefits can only be expressed through complex, application-specific life cycle cost analyses.

#### 6.2.2. Durability in Agricultural Environments

Ensuring long-term performance in the harsh agricultural environment is crucial, particularly for biocomposites, which face several durability challenges. Natural fibers are extremely hydrophilic and, hence, prone to water absorption when exposed to humidity, rain, soil moisture, and washing cycles (Table 1). Water absorption can cause the swelling of fibers, instability in dimensions, degradation of the fiber–matrix interface, and loss of mechanical properties such as strength and stiffness [98]. Additionally, moisture adsorption increases susceptibility to microbial attacks including rot and mildew. While moisture protection through matrix choice, fiber treatments, and coatings is necessary, it adds complexity and cost [42,43].

Biocomposites are also vulnerable to UV degradation (Table 1). Long exposure to sunlight can degrade the polymer matrix and lignin in natural fibers. UV stabilizers or protective coatings are often required for exterior components to ensure both durability and aesthetics [110,172]. Temperature sensitivity is also a concern, as natural fibers tend to have lower thermal degradation temperatures (typically below 200–230 °C, Table 1) and are thus limited from exposure to heat sources such as engines or exhaust systems in farm equipment. In addition, some bio-matrices such as PLA have very low heat distortion temperatures (~50–60 °C) and are thus not acceptable for use under sunlight or operating heat conditions. Therefore, the components of agricultural machines must be designed to run reliably over a broad range of environmental temperatures, from below freezing to hot summer days.

#### 6.2.3. Damage Tolerance, Inspection, and Repair

Composite materials exhibit different behaviors when impacted compared to metals. While they are effective energy absorbers, damage mechanisms such as matrix cracking, fiber failure, and, more importantly, delamination (separation of layers) can develop, which on occasion may not be evident on the surface but can significantly decrease the compressive strength and stiffness [130]. Repair techniques for composites, such as patching or resin infusion, are available but tend to be more complex than traditional metal repairs. These methods often require specialized materials and skills and may not fully restore the original strength and durability of the component, unlike welding or patching in metals [173]. This poses a critical challenge for AME, which frequently experiences rough handling, undergoes impacts from stones or tools, and often operates in remote locations where specialized repair facilities may not be readily accessible.

#### 6.2.4. Manufacturing and Consistency

Achieving consistency in part quality and mechanical performance remains one of the major challenges in composite material usage, especially for structural and load-bearing components [174,175]. Process variability is one of the primary concerns. Manual processes including hand lay-up are highly operator-skill and operator-experience dependent, resulting in variations in fiber placement, resin distribution, and quality of cure [36,53,102]. Despite the utilization of semi-automated or automated processes, strict control of process parameters—i.e., temperature, pressure, and resin flow—needs to be exerted to exclude common manufacture defects such as voids, incomplete impregnation, or undercured areas [53,91,112].

Another significant challenge is material variability, particularly for natural fibers. The mechanical properties of natural fibers can be wide-ranging due to differences in plant species, growing conditions, time of harvest, and methods of fiber extraction and processing [64,66]. This natural scatter complicates the design of reliable components and often requires rigorous quality control measures and conservative safety factors to be adopted to provide consistency in performance.

Scalability is another major challenge. The step from lab-scale or prototype production to industrial-scale manufacturing for AME applications needs robust, automated, and reproducible processes [11,20]. These must be developed to fit the specific requirements of composite materials, such as gentle fiber handling, exact resin dosing, and well-controlled curing cycles [10,13]. Achieving this level of manufacturing scale often involves high capital outlay and specialist technical expertise [176], which may limit the adoption of composite technologies among small- and medium-sized manufacturers.

#### 6.2.5. Standardization and Long-Term Data

The relatively new utilization of composites—particularly biocomposites—in AME applications has created a void for mature industry-specific design codes, standardized test protocols, and extensive long-term field performance information in comparison with conventional metals [159,167]. The absence of standardization introduces uncertainty that can diminish designer confidence, delay regulatory approval, and complicate the prediction of long-term durability, reliability, and maintenance requirements [169]. These challenges can be overcome through collective research, testing, and field validation to establish a solid foundation for the general application of composite materials in AME use.

#### 6.2.6. Fiber–Matrix Adhesion

Establishing a strong and durable bond between inherently hydrophilic (water-attracting) natural fibers and typically hydrophobic (water-repelling) polymer matrices remains a core scientific challenge in biocomposite development [26,99]. Inadequate adhesion compromises stress transfer across the interface, leading to reduced mechanical performance—particularly in terms of strength and toughness—and creates pathways for moisture ingress, which accelerates degradation over time [99,160]. Addressing this issue necessitates the use of effective, economical, and environmentally friendly fiber surface treatments, or the incorporation of coupling agents or compatibilizers into the matrix to enhance interfacial compatibility.

## 7. Future Directions and Research Recommendations

To fully unlock the potential of composite and biocomposite materials in the AME sector, targeted research and development efforts are required. These efforts should be aligned with the performance, durability, economic, and sustainability demands of modern agricultural practices. Key recommended areas of focus include the following:

- Development of advanced material systems.

Research should prioritize the creation of cost-effective, environmentally friendly treatments tailored to enhance the moisture resistance of natural fibers. These treatments must maintain performance under dynamic loads and harsh agricultural conditions such as exposure to rain, soil, and humidity. Investigating alternative natural fiber sources or genetically optimized crops could yield materials with superior intrinsic properties for AME applications.

Continued innovation in bio-based thermosets and thermoplastics is essential. These polymers should offer improved thermal stability (for proximity to engines), superior mechanical strength (for load-bearing structures), and enhanced processability and moisture resistance. Their performance should rival or exceed conventional petroleum-based resins used in current AME components.

The incorporation of nanomaterials—such as nanoclays, nanocellulose, and carbon nanotubes—into composites used in AME should be explored for their potential to significantly improve mechanical strength, chemical barrier properties, and wear resistance. These enhancements are particularly valuable for components such as sprayer tanks, fuel lines, and soil-engaging tools.

- Long-term durability assessment in realistic agricultural environments.

Systematic studies that combine accelerated laboratory weathering with real-world field tests are necessary. These must simulate UV exposure, moisture cycles, temperature fluctuations, contact with agrochemicals, and mechanical stresses typical of farm operations, enabling accurate service life predictions for composite AME parts.

Advanced computational models must be developed and validated to predict damage initiation (e.g., impact or tool strike) and propagation (e.g., fatigue cracking or delamination) in AME components. These models should reflect the complex static, dynamic, and fatigue loading patterns observed in actual farming scenarios.

- Sustainable and efficient manufacturing processes.

The development of cost-effective, automated manufacturing techniques—such as automated tape laying, resin transfer molding, and robotic lay-up—tailored for AME production volumes is essential. The parameter optimization of natural fiber composites in processes such as injection molding can also enhance part quality and reduce cost.

The use of 3D printing for biocomposites should be explored, particularly for rapid prototyping, low-volume part fabrication, and on-demand production of customized components. Additive manufacturing could also offer localized repair solutions, especially in remote agricultural regions.

- Circular economy strategies in agricultural applications.

Scaling up economically viable recycling solutions for thermoset composites (e.g., using solvolysis or pyrolysis) and developing efficient reprocessing methods for thermoplastics and biocomposites from retired AME parts is crucial.

Integrating circular economy principles at the design stage—such as making composite components easy to separate from metallic frames—can streamline both repairs during service life and material recovery at end-of-life.

For niche applications where biodegradability is functionally desirable (e.g., temporary soil-contacting parts such as mulch film holders, planting aids), biocomposites with reliable degradation rates under known soil conditions should be developed. This ensures functionality during use and safe decomposition afterward.

- Hybridization and smart structures.

Composite structures that strategically combine different materials (e.g., integrating metal reinforcements into composite frames, using CFRP strategically within GFRP sprayer booms, combining conventional composites with biocomposites) can optimize strength, weight, and cost-effectiveness for targeted AME functions.

Embedding sensors (e.g., fiber-optic or piezoelectric) into critical AME structures such as chassis components or large booms can enable the real-time monitoring of stress, strain, and early damage. This would support predictive maintenance, reduce unexpected breakdowns, and enhance equipment reliability during critical operations.

## 8. Conclusions

Composite and biocomposite materials hold excellent potential to enhance the design, performance, and sustainability of agricultural machinery and equipment. Conventional fiber-reinforced polymers (FRPs), and more specifically glass fiber-reinforced plastics (GFRPs), have been successful in lightweighting, corrosion resistance, and enabling innovative design in components such as body panels, tanks, and increasingly in large sprayer booms where lightweighting is vital. Carbon fiber-reinforced polymers (CFRPs) provide additional weight savings and performance, though at higher cost.

Biocomposites utilizing natural fibers or bio-based resins provide a promising avenue for improving agricultural sustainability and circular economy conformity. While offering low density and reduced environmental impact, they face challenges related to moisture and UV sensitivity, variable mechanical performance, fiber–matrix adhesion, and overall cost competitiveness.

The selection of the optimal material for any agricultural application entails an integrated consideration of structural demands, environmental conditions, weight targets, manufacturing processes, cost limits, and end-of-life management. In many cases, hybrid solutions employing conventional and bio-based materials may offer the most suitable combination of performance and sustainability.

Future advancements depend on focused research and development to overcome current barriers. The priorities include improving the durability and reliability of biocomposites, scaling up cost-effective production techniques, establishing viable recycling and disposal pathways, generating long-term performance data, and instituting industry-specific standards. Advances in these fields will be of paramount importance to the complete realization of the potential of composites and biocomposites to produce lighter, more efficient, and sustainable agricultural machinery capable of contributing to future global demands.

## Figures and Tables

**Table 1:** General comparison of material properties relevant to agricultural machinery [66,156,157,158,159,160,161,162,163,164].

Property	Steel	Aluminum	GFRP (Glass/Epoxy-VE)	CFRP (Carbon/Epoxy)	NFRP (e.g., Flax/PP) *	Biocomposite (e.g., Flax/PLA) *
Density (g/cm^3^)	~7.8	~2.7	~1.8–2.1	~1.5–1.6	~1.1–1.4	~1.2–1.4
Specific strength	Low	Medium	High	Very High	Medium	Low-Medium
Specific stiffness	Low	Medium	High	Very High	Medium	Low-Medium
Corrosion resistance	Poor	Good	Excellent	Excellent	Fair-Good	Fair-Good
Chemical resistance	Fair-Good	Fair-Good	Good-Excellent	Excellent	Fair-Good	Fair
Moisture absorption	None	Low	Very Low	Very Low	High	High
UV resistance	Excellent	Excellent	Good	Good	Poor-Fair	Fair-Poor
Max service temp (°C)	>500	~200–300	~100–200	~150–250	~80–120	~50–60
Recyclability	Excellent	Excellent	Difficult	Difficult	Fair–Good	Good
Environmental impact	High	High	Medium–High	Medium–High	Low-Medium	Low-Medium
Cost	Low	Medium	Medium	High	Low-Medium	Low-Medium

* Properties of natural fiber composites (NFRPs) and bio-based composites are highly variable depending on fiber type, treatment, matrix, processing, and fiber content.

## Data Availability

The original contributions presented in this study are included in the article. Further inquiries can be directed to the corresponding authors.
